# Red blood cell alloantibodies in paediatric transfusion in sub‐Saharan Africa: A new cohort and literature review

**DOI:** 10.1002/jha2.645

**Published:** 2023-02-09

**Authors:** Macoura Gadji, Guéda Cobar, Alioune Thiongane, Alioune Badara Senghor, Rose Seck, Blaise Félix Faye, Moussa Seck, Youssou Bamar Guéye, Diariétou Sy, Abibatou Sall, Awa Oumar Toure, Tandakha Ndiaye Diéye, Saliou Diop

**Affiliations:** ^1^ Service of Haematology and Oncology‐Haematology (HBOH) Department of Biology and Applied Pharmaceutical Sciences Faculty of Medicine Pharmacy and Odonto‐Stomatology (FMPOS) University Cheikh Anta Diop of Dakar (UCAD) Dakar Senegal; ^2^ National Centre of Blood Transfusion (CNTS) Dakar Senegal; ^3^ Service of Paediatrics Department of Medicine, Hospital Albert Royer of Fann Faculty of Medicine Pharmacy and Odonto‐Stomatology (FMPOS) University Cheikh Anta Diop of Dakar (UCAD) Dakar Senegal; ^4^ Service of Haematology Department of Medicine, Faculty of Medicine Pharmacy and Odonto‐Stomatology (FMPOS) University Cheikh Anta Diop of Dakar (UCAD) Dakar Senegal; ^5^ Service of Biology Hospital Aristide le Dantec Dakar Senegal; ^6^ Service of Immunology Department of Biology and Applied Pharmaceutical Sciences Faculty of Medicine Pharmacy and Odonto‐Stomatology (FMPOS) University Cheikh Anta Diop of Dakar (UCAD) Dakar Senegal

**Keywords:** alloimmunization, blood transfusion, irregular antibodies, paediatrics, red blood cells

## Abstract

Blood transfusion support predisposes transfused children to the risk of erythrocyte alloimmunization in Sub‐Saharan Africa. A cohort of 100 children receiving one to five blood transfusions were recruited for screening and identification of irregular antibodies using gel filtration technique. The mean age was 8 years and the sex‐ratio at 1.2. The retrieved pathologies were: major sickle cell anaemia (46%), severe malaria (20%), haemolytic anaemia (4%), severe acute malnutrition (6%), acute gastroenteritis (5%), chronic infectious syndrome (12%) and congenital heart disease (7%). The children presented with haemoglobin levels ≤6 g/dl, and 16% of them presented positive irregular antibodies directed against the Rhesus (30.76%) and Kell (69.24%) blood group systems. A literature review shows that irregular antibody screenings vary from 17% to 30% of transfused paediatric patients in Sub‐Saharan Africa. These alloantibodies are in particular directed against the Rhesus, Kell, Duffy, Kidd and MNS blood group and generally found in sickle cell disease and malaria. This study highlights the urgent need of extended red blood cell phenotyping including typing for C/c, E/e, K/k, and Fya/Fyb, and if possible Jka/Jkb, M/N, and S/s for children before transfusion in Sub‐Saharan Africa.

## 1 INTRODUCTION

Blood transfusion is a supportive therapy, which is abusively used in Africa [1, 2, 3, 4] and in other low‐ and Middle‐income countries (LMICs), especially in paediatrics and obstetric services to treat severe and poorly tolerated anaemia [1, 5–7]. Blood transfusion is usually the first line therapy to severe anaemia and represents a cornerstone in medical practice in Africa allowing to reduce morbidity and mortality in paediatrics and obstetric services, in particular. Indeed, Africa and other LMICs face genetic and/or chronic or tropical diseases that usually require transfusion supports due to late diagnosis, and their negative impact in circulating blood [8, 9]. Transfusion support requires a rigorous process and medical rules before realization which are often weakly followed in LMICs as in Sub‐Saharan Africa (SSA). Despite its major medical usefulness as a therapeutic option, transfusion supports might be only used as a last resort because it is never without biohazards and other threats [1, 4, 10]. Indeed, blood transfusion brings donor antigens to the blood recipient which can cause immediate intolerances or late transfusion events leading to life threatening [10]. To minimize these transfusion biohazards, two tests are generally requested before any blood transfusion;—a first test as red blood grouping of donor and recipient to determine their ABO blood group type and RhD status.—a second test is cross‐matching donor red cells and recipient plasma or serum to ensure the compatibility of the donor and recipient [10] and/or a laboratory compatibility test [11]. Both tests are strictly followed in high level income countries (HICs) but weakly or not followed at all in some LMICs in SSA. During each transfusion, the rules of immunological compatibility must be taken into account because the most compatible transfusions are isogroup. Blood transfusion support in SSA is generally performed in ABO and RhD blood group compatibility without further determinations in other red blood group systems. It is then obvious that (multi)‐transfused patients are always prone to the risk of alloimmunization with respect to other erythrocyte antigens [11, 12]. The major adverse effect of blood transfusion is alloimmunization e.g., formation of irregular antibodies (IAs) to non‐self‐antigens on red blood cells of donors. The incompatibility between the blood donor and recipient determines the level of immunological risks of alloimmunization [10, 12]. This erythrocyte incompatibility generates formation of IAs which can be responsible for transfusion accidents or incidents associated with a subsequent consequence of transfusion inefficiency. This alloimmunization results from immune system stimulation that increases the risk of haemolytic transfusion reactions and leads to delays in identification of compatible red blood cell units [12]. These haemolytic accidents are generated by the appearance of IAs against blood group antigens on the transfused red blood cells of the donor unknown to the recipient. In addition, these alloimmune antibodies appear to be the most complicated situation in haemolytic transfusion reactions. However, the effect of providing an antigen takes precedence over that of an antibody [10, 13], which may even be negligible. However, this situation may turn out to be different, especially if the antibody is supplied at a high titer as in multi‐transfusion in SSA, usually. These immunological risks remain poorly assessed in SSA and are not always avoided, especially when the transfusion support is intended for multi‐transfused patients such as paediatric patients or pregnant women [7, 11, 13].

In fact, this alloimmunization depends on the number of transfusions, the immune status of the recipient and the antigenic differences between blood donor and recipient. While in HICs, screening for IAs is a systematic part of preventing transfusion complications, this is not the case in LMICs as SSA, generally.

In order to evaluate the immunological safety of blood transfusion support in SSA, we set out to screen and identify the presence of IAs in hospitalized and transfused sick children in one of the biggest paediatric hospital of the country and in SSA. Furthermore, a review of the medical literature in SSA (due to limited available publications) was performed aiming to highlight the negative impact of these IAs in the safety and efficiency of blood transfusion support in sick paediatric patients in this part of the World.

## 2 MATERIALS AND METHODS

### 2.1 Study population

Hundred patients were recruited as they were hospitalized at the paediatric hospital Albert Royer at the National University‐Hospital of Fann for various diagnosed pathologies. This study was approved by the Research Ethic Comity of the Cheikh Anta Diop University of Dakar (Senegal) (0415/2019/CER/UCAD). After informed oral and/or written consent of the parents (written when alphabet parents, oral and written when analphabetic parents), venous blood sampling was performed from sick children whom medical treatment required blood transfusion therapy.

### 2.2 Inclusion criteria

Any hospitalized paediatric patient at the paediatric hospital who has received at least one transfusion support of red cell concentrates or more and after informed consent of the parents.

### 2.3 Non‐inclusion criteria

Newborns and children without informed parental consent were excluded from the study or their medical treatment did not required blood transfusion support.

### 2.4 Sampling

Blood samples were taken by venous phlebotomy from each child whose last transfusion support was done 10–15 days ago. Venous blood were collected on dry or EDTA tubes and the serum or plasma was stored at ‐20°C until use for irregular red blood cell antibody screening and identification.

### 2.5 Screening and identification of irregular agglutinins

The method with gel filtration cards was used to screen and identify irregular agglutinins [14]. The used panels, reagents and low ionic strength solution (LISS) /Coombs gel cards are all from the DiaMed laboratory (Diamed, Murten, Switzerland). The Gel cards are:—ID Micro Typing Cards LISS/Coombs‐Enzyme for antibody screening;—ID Micro Typing Cards LISS/Coombs and ID Micro Typing Cards NaCl/Enzyme for identification. The panel of red cells for screening and identification are: ‐ID DiaCell I+II+III (three non‐papainized cells at 0.8%) and ID DiaCell I+II+IIIP (three papainized cells at 0.8%) for screening;—ID DiaPanel (11 non‐papainized cells at 0.8%) and ID DiaPanelP (11 papainized cells at 0.8%) for identification.

**FIGURE 1:**
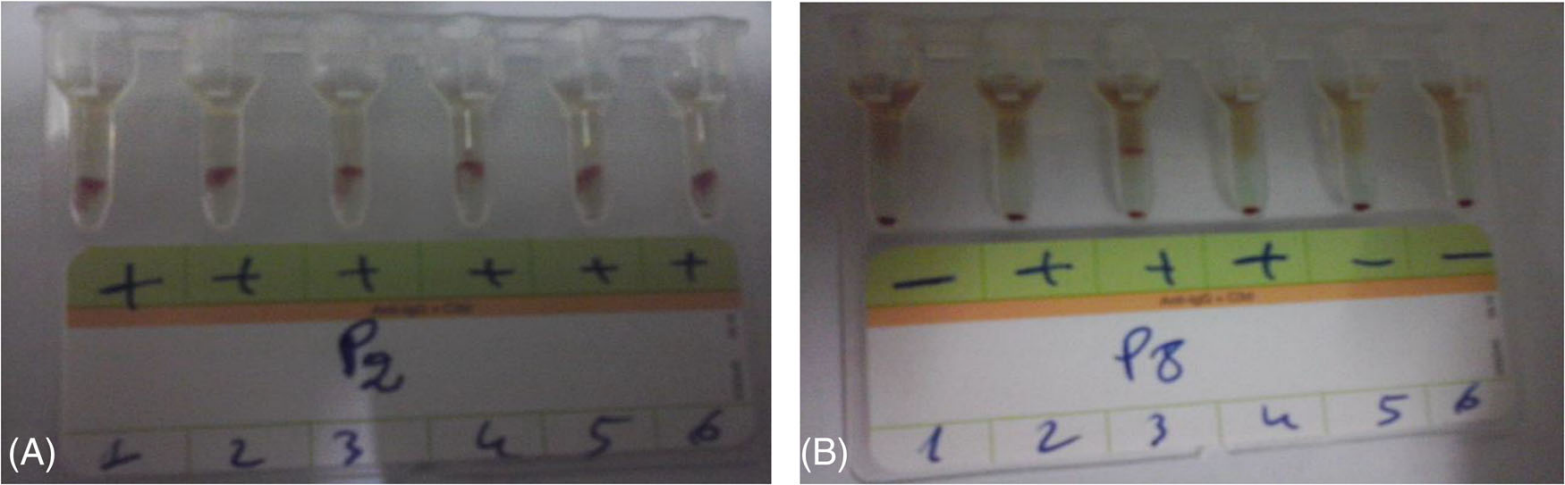
Screening and identification of irregular antibodies on low ionic strength solution (LISS)/Coombs gel filtration. A = Example of a positive IAs result on all wells. B = Example of negative IAs result on wells 1, 5 and 6

The detection of an IA by gel filtration card is based on red blood cell antigen‐antibody reactions which develop in a special gel on which the tested red blood cells and the serum samples are deposited. These reactions occur after a 10 min centrifugation at 80 g (910 RPM) in a ID centrifuge (Diamed, Murten, Switzerland) and is revealed by haemagglutination reactions trapped on top and/or inside the gel (Figure 1). The screening was performed using the screening cards combining indirect Coombs and papain techniques using the respective sets of three red cells. The identification of IAs was performed with the aid of the panel of 11 red cells.

### 2.6 Data collection and analyses

For each paediatric patient, a recruitment form was completed collecting epidemiological and clinical data. Excel 2010 software was used for data processing and analyses were performed using EPI INFO 6.

## 3 RESULTS

### 3.1 Clinical and epidemiological parameters of the population

The population consisted of 100 child patients, 55% male and 45% female. The average age was 8 years with extremes ranging from 3 months to 15 years; 70% of the children were between 6 and 8 years old in which 50% were male. The children presented with severe and poorly tolerated normochromic normocytic anaemia in 90% of cases. They all had a haemoglobin level ≤ 6 g/dl.

**FIGURE 2:**
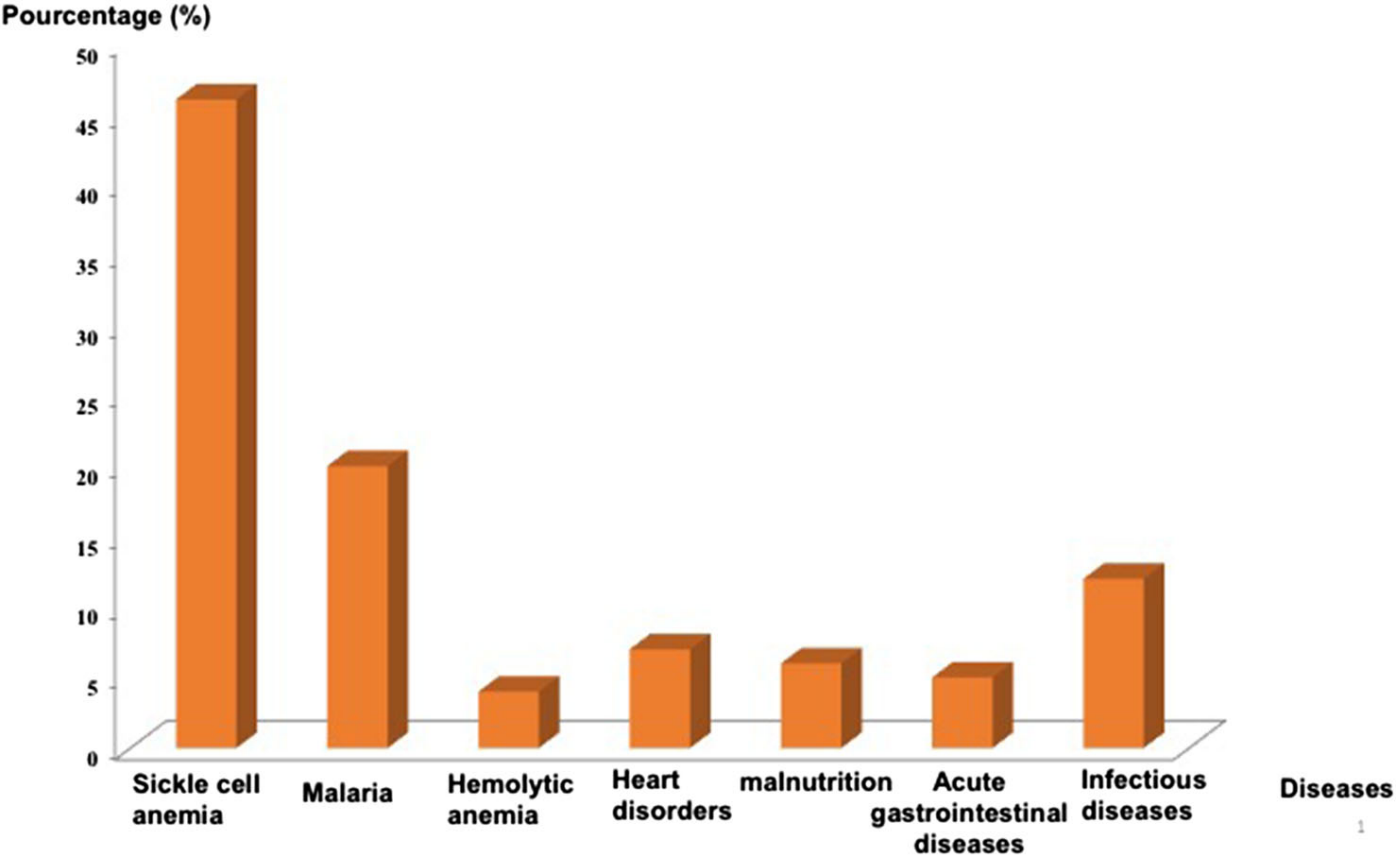
Frequency of identified diseases in the paediatric population

Clinically, seven pathologies were identified, of which sickle cell disease (SCD) is the most common (46%) followed by malaria (20%), infectious syndromes (12%), congenital heart disease (7%), severe acute and complicated malnutrition (6%), acute gastroenteritis (AGE; 5%) and haemolytic anaemia (4%) (Figure 2).

ABO and RhD blood group phenotypes were also determined, but there was no RhD negative. Thus, 20% of the children in this study were in the A RhD positive group, 15% in the B RhD positive group, 56% in the O RhD positive group and 9% in the AB RhD positive group.

### 3.2 Prevalence of IAs

The screening of IAs showed that 16 paediatric patients (16%) were positive for IAs, the majority of them received a maximum of 5 blood transfusions.

### 3.3 Identification of screened IAs

The identification of IAs was performed in 13 paediatric patients and 5 types of antibodies were detected with a predominance of Ab anti Kell (Tables 1 and 2). In addition, 2% of children showed anti Rh antibodies. Because of insufficient blood samples the remaining 3 child were not identified.

**TABLE 1:** Identification of irregular antibodies in the paediatric population

Antibodies	Number of positive patients	Percentage
Anti D	02	2
Anti C	01	1
Anti c	01	1
Anti Kell (anti K, anti kp^b^)	09	9
Not performed	3	3
TOTAL	13	16

**TABLE 2:** Short list (non‐exhaustive) of examples of IAs in LMICs versus HIC

SSA Countries	Number, age and sex of the studied paediatric population	IAs (%)	Ab specificities	References
Senegal	253 SCD patients including 56 patients ≤ 20 years	16	anti‐Rhesus (34.19%),	[61]
	Mean age = 28.5 years with extremes 5‐59 years;		anti‐Kell (23.67%),	
	sex ratio = 0.86		anti‐Duffy (10.52%),	
			anti‐Kidd (2.63%),	
			anti‐Lewis (5.26%),	
			anti‐Lutheran (18.41%)	
Mali	78 patients	10.3	anti‐E (7.7%)	[13]
	Mean age : 36.78 ± 14.73 years with extremes 11‐77 years.		anti‐C (1.3%)	
	Sex‐ratio: 1.11		anti‐D (1.3%)	
Democratic Republic of Congo (DRC) (Kisangani)	125 SCD patientsMedian age: 15.5 years with extremes 4.8–19.8 for alloimmunized patients	9.6	anti‐D (28.6%) anti‐C	[33]
	Median age: 24 years (IQR:14–31) for non‐allo immunized patients			
Belgique The CHR de la Citadelle of Liège	136 SCD patients at Median age: 10 years with extremes: 6.5–17 years for allo immunized patients	22.8	anti‐E (13.6%), anti‐S (13.6%) and anti‐Lea (11.4%)	
	Mean age:17 years with extremes 12–24 years for non‐allo immunized patients			
Ivory Coast	86 SCA patients	26.74	52.18% of IgG type	[53]
	age: 1 to 40 years		21.74% of complement type	
			17.39% mixed IgG and complement types	
Nigeria	145 SCA subjects	9.3	anti‐E (37.5%)	[45]
	mean of 26 ± 7.4 years with extremes 18‐48 years		anti‐C (25%)	
	73 males (50.3%) and 72 females (49.7%)		anti‐D (12.5%)	
			anti‐e (12.5%)	
Morocco	96 patients	17.07	32% anti‐E,	[34]
	Median age: 11 years with extremes 2‐28 years		10% anti‐C,	
	Male 53%		5% anti‐c,	
	Female : 47%		22% anti‐K,	
			16% anti‐Jka,	
			5% anti‐Fya,	
			5% anti‐Fyb	
			5% anti‐Lua.	
France	1575 patientsMedian age: 63.5 years Sex‐ratio: 3.03	15	RH3/E (18.7%), KEL1/K (17.3%), RH1/D (16.4%), MNS1/M (9.4%), FY1/Fya (6.9%), RH2/C (6.1%), KEL3/Kpa (4.7%), JK1/Jka (4.3%) RH4/c (4.1%).	[58]
	173 SCA patients (59 transfused exclusively with frozen red blood cells (group 1)	Group 1: 8.2	Abs against Jkb, Jka, Fya and S; 1 patient developed 2 antibodies.	
	and			[56]
	124 patients who received standard red blood cells (group 2)	Group 2 : 30.6	Abs against C, E, K, Fya; 16% developed antibodies reacting with different antigens	

### 3.4 Distribution of irregular antibodies versus diseases

**FIGURE 3:**
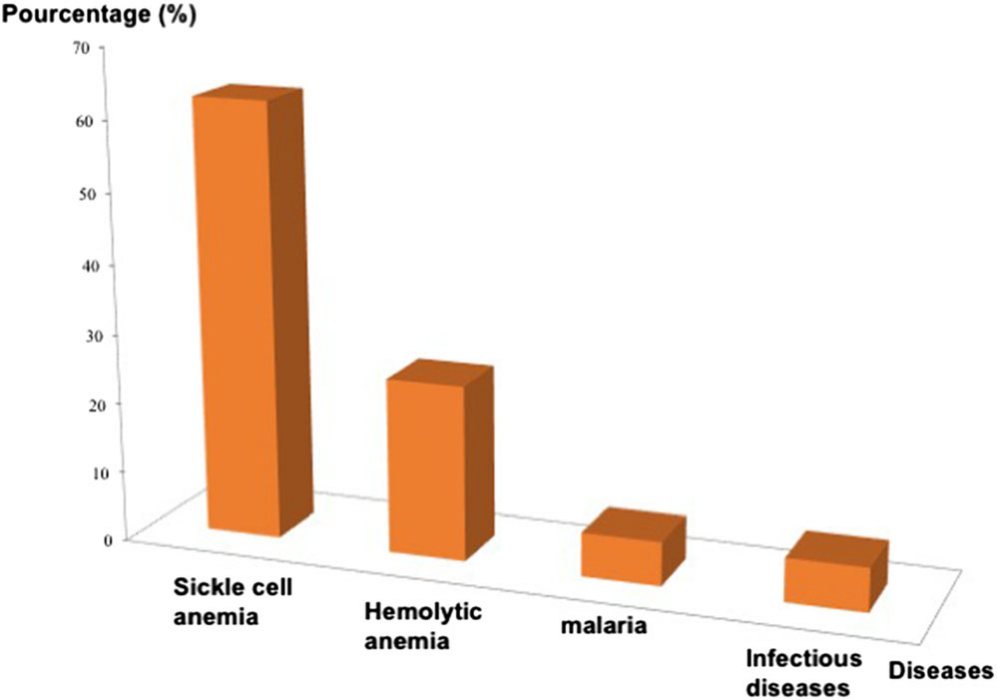
Repartition of irregular antibodies versus diseases of paediatric population

IAs have been detected only in four pathologies; sickle cell disease (SCD) was the most prevalent followed by haemolytic anaemia, malaria and infectious diseases. An absence of IAs was noted in other pathologies as congenital heart disease, severe acute and complicated malnutrition, acute gastroenteritis (Figure 3).

### 3.5 Distribution of IAs versus ABO and RhD blood groups

The majority of IAs was detected in children of the O RhD positive blood group (56.25%). The remainder of the IAs occurred in children with A RhD positive (25%) and B RhD positive (18.75%) blood groups. No IA was detected in AB RhD positive blood group of children.

## 4 DISCUSSION

Blood transfusion support represents a cornerstone for medical practice aiming to improve oxygen‐carrying capacity and symptoms of anaemia. In daily medical practice, transfusion support requires high standards for infectious and immunologic safety which are allowed in HICs. However, in LMICs where resides around 80% of the world's population, only 20% of the worldwide supply of safe and screened blood is available according to WHO [4, 10, 15, 16, 17, 18, 19, 20]. Despite the improvements on transfusion safety over a few decades in LMICs in SSA, these countries continue to struggle with inadequate resources and infrastructure for a safer blood supply [10, 15, 17, 20]. Thus, in SSA, the dilemma is an important need of blood transfusion to treat severe and chronic anaemia resulting from tropical diseases contrasting with low or moderate safety of transfusion [15, 16, 17, 19, 20]. Indeed, a higher rate of transfusion is routinely performed for recipients typically including sick children, pregnant women, victims of trauma, sickle cell disease patients, persons with other haemoglobinopathies, severe parasitic infections, and nutritional anaemia [21, 22, 23, 24, 25, 26, 27, 28, 29].

### 4.1 Epidemiological characteristics of transfused paediatric patients in SSA

In Africa, 19% to 67% of hospitalized children receive transfusion, and children constitute the largest proportion of transfusion recipients [18, 21, 22, 23, 30, 31]. Children included in this study are from 3 months to 15 years old with a mean age of 8 years, whereas in Uganda, 65% of all transfusions are performed on children under age 5 [30, 32]; in Kenya 58% of transfusions were for children under age of 10 years and 37% for children under age 2 [31]; in Democratic Republic of Congo 9.6% [33]; and in Mali and Morocco an average age of 11 years were reported [13, 34].

### 4.2 IAs in transfused paediatric patients in SSA

It is obvious that children constitute a group at risk of alloimmunization due to frequent use of transfusion, despite that simple transfusions are dosed by volume (*i.e*.,10‐15 mL/kg) in paediatric patients [23, 24, 25, 26]. Indeed, most of the blood banks in SSA perform only ABO grouping and Rh typing and no cross‐matching test, neither additional alloantibody or auto‐antibody screening and identification are performed before and after transfusion [35, 36, 37, 38]. This might be related to limited resources obliging to lower the cost of transfusion practice, but also to limited technical expertise, lack of automation, reagents, and adequate training [1, 4, 10, 15, 18, 19, 20]. Thus, multi‐transfused paediatric populations are exposed to alloimmunization e.g., production of IAs to foreign antigens. It is of note that transfusion alloimmunization can appear years after an immunizing transfusion and compromise the future transfusion outcome of the patient especially in children and pregnant women [7, 8, 9, 10, 11]. As a major adverse effect of blood transfusion, transfusion alloimmunization augments the risk of haemolytic transfusion reactions and leads to delays in identification of compatible red cell units [11, 12, 29, 40, 41]. All degrees of severity can be observed from the immediate haemolytic event complicated by renal failure [2, 5, 8, 36]. That is why, in many developed countries, IAs are systematically screened [38, 42, 43, 44] before any transfusion in any patient likely to be transfused in the short term, and 10–15 days after each transfusion, in multi‐transfused patients [3, 5, 10, 11].

### 4.3 Relationship between IAs and diseases in paediatric patients in SSA

Children in this study presented with severe normochromic, normocytic anaemia that was poorly tolerated with haemoglobin levels below 6 g/dl before a new transfusion. A high frequency of IAs in paediatric patients was observed related to blood transfusion in which the leading reason of transfusion was sickle cell disease (SCD) followed by malaria‐associated anaemia. Both diseases share early clinical manifestations predisposing children to a life time blood transfusion support becoming multi‐transfused patients with a high risk of producing IAs. In Uganda, the majority of transfused sick children suffer from malaria and other infections, malnutrition, and SCD [30, 32]; in Kenya paediatric transfusions were largely performed for malaria (49%), followed by surgery (14%) and chronic anaemia (11%) [21, 22, 31]; while malaria is the major indication transfusion therapy in Mozambique [45].

Despite overall improvements in blood inventory and safety in the last decades, adverse effects of transfusion are prevalent among paediatric patient with sickle cell disease in Africa [35, 42, 43, 44, 46, 47]. These adverse effects include alloimmunization generating diverse IAs, acute and delayed haemolytic transfusion reactions, and iron overload [35, 37, 38, 42, 44]. Indeed, patients with SCD are the largest population group requiring multiple transfusions. Most of the world's population with SCD resides in Africa, with the remainder in the Middle East, Caribbean, or India [38, 42, 46]. Thalassemia, an important public health problem in Southeast Asia, Africa, India, and the Middle East, constitutes another major population group that receives a large proportion of blood transfusions in LMICs. Several studies confirmed sickle cell anaemia and malaria‐associated anaemia as the major indications of blood transfusion support in African services of paediatrics [11, 23, 24, 48]. However, the reasons for hospitalization were mainly chronic renal failure followed by major SCD (HbSS) according to other study [27, 44, 49].

### 4.4 Frequencies and specificities of IAs in paediatric patients in SSA

The identified IAs in this study, were antibodies mostly directed against the Rhesus and the Kell systems. The most prevalent IAs detected are anti D (15.38%), anti c (7.69%), anti C (7.69%) and anti‐Kell (k + kpb) (69.24%). In Nigeria, the prevalence of red cell alloantibody among multi‐transfused patients with SCD was found to be 9.3% [46]. Alloantibodies identified were mainly against Rhesus antigens contributing 87.5% and a combination of Kell and Lutheran blood group antigens contributed 12.5% [46]. A meta‐analyse study showed that a pooled proportion of alloimmunization in SCD in SSA was 7.4% with almost 50% of antibody specificities were against D, C, E, and K antigens [47]. Also, the antibodies to low‐ and high‐frequency antigens accounted for 29% of antibodies [47] while in a previous meta‐analyse study an overall proportions of alloimmunization were 6.7% of transfused patients [37]. With regard to antibody specificity, among clinically significant antibodies, anti‐E ranked as the most common, followed by anti‐K, anti‐C and anti‐D [37]. The presence of anti‐D antibodies in the RhD positive studied children populations might be related to absence or lack of a proper anti‐RhD therapy during potential alloimmunization and/or to different potential variants in the Rhesus system related to African genetic background. Indeed, despite serological matching for D and C, E or C/c, E/e antigens, Rh alloimmunization persists due to Rhesus genetic diversity in individuals of African descent. The *RHD* and *RHCE* genes are located on chromosome 1, arose through gene duplication, and encode D and C, c, E, e antigens, respectively. The two loci are highly homologous, leading to many gene recombination events resulting in variant *RHD* and *RHCE* alleles that encode altered antigens [50, 51, 52].

Several authors in SSA also reported high frequencies of these antibodies in post‐transfusion erythrocyte alloimmunization [11, 13, 21, 32, 33, 41, 46, 53, 54]. In Mali, different series were reported with a frequency of 10.3% in a study population in which eight antibodies were identified, all anti‐Rhesus [13] and a frequency almost similar to ours, *i.e*. 17.39% in a second study [55]. In Ivory Coast, a study showed an incidence of post‐transfusion anti‐erythrocyte alloimmunization to the SCD patients was of 26,74% [54]. In Marocco, a study carried out on 96 patients, showed an alloimmunization frequency of 17.07% similar to our results [34]. These detected antibodies were often directed against erythrocyte antigens of the Rhesus, Kell, Duffy and Kidd systems [34].

Similar frequencies were observed in different studies in France in Guadeloupe (10.8%) [56] and in Creteil (8.2%) [57]. Another study was carried out on a population of polytransfused SCD in France, found a frequency of 15% positive IA [58]. In addition a retrospective study carried out to analyse the IAs identified in 2008 [58] in the same country showed that the most frequent alloantibodies were directed against the Rhesus and Kell systems [59]. In general, the proportion of transfused sickle cell anaemia patients varies from different studies, between 30% and 90% [60]. This variation can be related to environmental factors, disease genetic factors and other factors including the low availability of blood, difficulties in accessing to health care and inadequacies of the transfusion system [60, 61]. Thus, it is obvious to understand that the British Society for Haematology (BSH) guidelines, the American Society of Haematology (ASH) 2020 guidelines, and the USA National Institutes of Health Expert Panel (NIHEP) recommend prophylactic matching for Rh (C, E or C/c, E/e), and K in addition to ABO and D in patients with SCD [42, 43, 44, 47].

Finally, IAs are an obstacle and a real public health problem in blood transfusion. They are responsible for serious medical consequences in (poly)transfused patients. It is therefore important to search for such antibodies before and after any transfusion in order to avoid these serious post‐transfusion events and especially the transfusion stalemate in polytransfused subjects such as children. Judicious use of red cell transfusions, optimization of red cell antigen matching, and the use of erythrocytapheresis and iron chelation can minimize adverse effects. Early recognition and management of haemolytic transfusion reactions can avert poor clinical outcomes [24, 25, 35, 37, 48].

## 5 CONCLUSION

This study shows a high frequency of IA in sick children in paediatrics. The frequency of IAs in SSA varies from 16 to 17% averaging 30% in some areas and mostly directed against the Rhesus, Kell, Duffy, Kidd and MNS blood group systems. All these IAs might be responsible for immunological reactions, transfusion inefficiency and above all can lead to transfusion stalemate. To decrease this alloimmunization frequency, it would be necessary to systematize the phenotyping of the blood of donors and of recipients by including typing for C/c, E/e, K/k, and Fya/Fyb at least, and Jka/Jkb, M/N, and S/s if possible. In addition, systemise screening for IAs before and after any transfusion is mandatory in SSA. Otherwise, these recommendations should be applied at least in paediatric patients at risk of multi‐transfusion therapy and in polytransfused population risk groups such as SCD patients as recommended by among others BSH, ASH and NIHEP.

## CONFLICT OF INTEREST

No conflict of interest to disclose
